# Supplementary material for: Plasma metabolomics profiling identifies new predictive biomarkers for disease severity in COVID-19 patients
Source: PLoS One. 2023 Aug 10;18(8):e0289738. doi: 10.1371/journal.pone.0289738 (PMC10414581; doi:10.1371/journal.pone.0289738)
Supplement: S1 Table — (DOCX) [file pone.0289738.s001.docx]

Supplementary Table 1. Statistical Significance of ROC AUC of metabolites in predicting severity of COVID-19

| **No** | **Metabolite test** | **AUC** | | **P-value** | **Cut-off*** | **Sensitivity** | | | **Specificity** | |
| --- | --- | --- | --- | --- | --- | --- | --- | --- | --- | --- |
|  |  | **Value** | **95% CI :LL to UL** |  |  | **Value** | **95% CI :LL to UL** | **Value** | | **95% CI :LL to UL** |
| 1 | K_1_3_Dimethyluric_acid | 0.167 | 0.070 to 0.263 | <0.001 | - |  |  |  | |  |
| 2 | K_1_Methyladenosine | 0.345 | 0.225 to 0.465 | 0.016 | - |  |  |  | |  |
| 3 | K_2_5_Furandicarboxylic_acid | 0.126 | 0.050 to 0.201 | <0.001 | - |  |  |  | |  |
| 4 | K_2_Pyrrolidinone | 0.452 | 0.328 to 0.575 | 0.444 | - |  |  |  | |  |
| 5 | K_3_4_5_Trimethoxycinnamic_acid | 0.226 | 0.125 to 0.326 | <0.001 | - |  |  |  | |  |
| 6 | K_3_5_Dimethoxyphenol | 0.494 | 0.370 to 0.619 | 0.930 | - |  |  |  | |  |
| 7 | K_3_Indolepropionic_acid | 0.213 | 0.109 to 0.318 | <0.001 | - |  |  |  | |  |
| 8 | K_3_Methylindole | 0.017 | 0.000 to 0.036 | <0.001 | - |  |  |  | |  |
| 9 | K_3_Methylxanthine | 0.095 | 0.026 to 0.164 | <0.001 | - |  |  |  | |  |
| 10 | K_4_Aminophenol | 0.883 | 0.803 to 0.962 | <0.001 | 381.5 | 82.2% | 67.4% to 91.5% | 90.0% | | 75.4% to 96.7% |
| 11 | K_5_Hydroxy_L_tryptophan | 0.048 | 0.007 to 0.090 | <0.001 | - |  |  |  | |  |
| 12 | K_5_Hydroxyindoleacetic_acid | 0.386 | 0.265 to 0.506 | 0.070 | - |  |  |  | |  |
| 13 | K_9_Methyluric_acid | 0.188 | 0.088 to 0.289 | <0.001 | - |  |  |  | |  |
| 14 | Acetaminophen | 0.949 | 0.894 to 1.000 | <0.001 | 1595.5 | 90.9% | 77.4% to 97.0% | 91.2% | | 75.2% to 97.7% |
| 15 | Acetaminophen_glucuronide | 0.791 | 0.539 to 1.000 | 0.015 | 1416.0 | 78.0% | 62.0% to 88.9% | 85.7% | | 42.0% to 99.2% |
| 16 | Acetic_acid | 0.612 | 0.490 to 0.733 | 0.077 | - |  |  |  | |  |
| 17 | Acetone | 0.459 | 0.334 to 0.585 | 0.520 | - |  |  |  | |  |
| 18 | Adenosine_monophosphate | 0.160 | 0.076 to 0.244 | <0.001 | - |  |  |  | |  |
| 19 | Allantoin | 0.459 | 0.335 to 0.583 | 0.518 | - |  |  |  | |  |
| 20 | Alpha_ketoisovaleric_acid | 0.022 | 0.000 to 0.047 | <0.001 | - |  |  |  | |  |
| 21 | Alpha_N_phenylacetul_L_glutamine | 0.524 | 0.400 to 0.648 | 0.698 | - |  |  |  | |  |
| 22 | Aniline | 0.411 | 0.285 to 0.537 | 0.156 | - |  |  |  | |  |
| 23 | Aspartame | 0.502 | 0.374 to 0.630 | 0.972 | - |  |  |  | |  |
| 24 | Azelaic_acid | 0.267 | 0.153 to 0.381 | <0.001 | - |  |  |  | |  |
| 25 | Benzaldehyde | 0.341 | 0.221 to 0.460 | 0.012 | - |  |  |  | |  |
| 26 | Benzocaine | 0.476 | 0.348 to 0.603 | 0.698 | - |  |  |  | |  |
| 27 | Benzoic_acid | 0.215 | 0.117 to 0.313 | <0.001 | - |  |  |  | |  |
| 28 | Cadaverine | 0.339 | 0.220 to 0.458 | 0.011 | - |  |  |  | |  |
| **No** | **Clinical /Metabolite test** | **AUC** | | **P-value** | **Cut-off*** | **Sensitivity** | | | **Specificity** | |
|  |  | **Value** | **95% CI :LL to UL** |  |  | **Value** | **95% CI :LL to UL** | **Value** | | **95% CI :LL to UL** |
| 29 | Caffeine | 0.159 | 0.074 to 0.245 | <0.001 | - |  |  |  | |  |
| 30 | Chlorpheniramine | 0.364 | 0.239 to 0.489 | 0.032 | - |  |  |  | |  |
| 31 | Cinnamic_acid | 0.347 | 0.226 to 0.468 | 0.015 | - |  |  |  | |  |
| 32 | Cis_Aconitic_acid | 0.193 | 0.101 to 0.284 | <0.001 | - |  |  |  | |  |
| 33 | Cortisol | 0.401 | 0.273 to 0.528 | 0.115 | - |  |  |  | |  |
| 34 | Creatine | 0.196 | 0.099 to 0.293 | <0.001 | - |  |  |  | |  |
| 35 | Cytosine | 0.784 | 0.680 to 0.887 | <0.001 | 818.0 | 68.2% | 52.2% to 80.9% | 90.0% | | 75.4% to 96.7% |
| 36 | Deoxycholic_acid_glycine_conjugate | 0.219 | 0.116 to 0.322 | <0.001 | - |  |  |  | |  |
| 37 | DL_2_aminooctanoic_acid | 0.117 | 0.042 to 0.192 | <0.001 | - |  |  |  | |  |
| 38 | Elaidic_acid | 0.641 | 0.521 to 0.761 | 0.025 | - |  |  |  | |  |
| 39 | Ethanolamine | 0.229 | 0.129 to 0.330 | <0.001 | - |  |  |  | |  |
| 40 | Glucosamine | 0.397 | 0.272 to 0.521 | 0.103 | - |  |  |  | |  |
| 41 | Glycerophosphocholine | 0.087 | 0.027 to 0.148 | <0.001 | - |  |  |  | |  |
| 42 | Glycine | 0.640 | 0.520 to 0.760 | 0.027 | - |  |  |  | |  |
| 43 | Glycocholic_acid | 0.340 | 0.222 to 0.458 | 0.012 | - |  |  |  | |  |
| 44 | Guanidine | 0.362 | 0.242 to 0.481 | 0.028 | - |  |  |  | |  |
| 45 | Hippuric_acid | 0.253 | 0.142 to 0.364 | <0.001 | - |  |  |  | |  |
| 46 | Homoveratric_acid | 0.503 | 0.379 to 0.627 | 0.961 | - |  |  |  | |  |
| 47 | Hypoxanthine | 0.205 | 0.106 to 0.305 | <0.001 | - |  |  |  | |  |
| 48 | Indole | 0.020 | 0.000 to 0.043 | <0.001 | - |  |  |  | |  |
| 49 | Indoleacetic_acid | 0.211 | 0.112 to 0.310 | <0.001 | - |  |  |  | |  |
| 50 | Indolelactic_acid | 0.142 | 0.054 to 0.230 | <0.001 | - |  |  |  | |  |
| 51 | Inosinic_acid | 0.136 | 0.061 to 0.211 | <0.001 | - |  |  |  | |  |
| 52 | Isobutyric_acid | 0.678 | 0.561 to 0.795 | 0.005 | - |  |  |  | |  |
| 53 | Isovalerylcarnitine | 0.402 | 0.275 to 0.528 | 0.119 | - |  |  |  | |  |
| 54 | Kynurenic_acid | 0.331 | 0.214 to 0.448 | 0.007 | - |  |  |  | |  |
| 55 | L_Acetylcarnitine | 0.374 | 0.251 to 0.498 | 0.047 | - |  |  |  | |  |
| 56 | L_Arginine | 0.062 | 0.012 to 0.112 | <0.001 | - |  |  |  | |  |
| 57 | L_Carnitine | 0.296 | 0.182 to 0.409 | 0.001 | - |  |  |  | |  |
| 58 | L_Glutamine | 0.527 | 0.402 to 0.652 | 0.674 | - |  |  |  | |  |
| 59 | L_Histidine | 0.090 | 0.032 to 0.148 | <0.001 | - |  |  |  | |  |
| **No** | **Clinical /Metabolite test** | **AUC** | | **P-value** | **Cut-off*** | **Sensitivity** | | | **Specificity** | |
|  |  | **Value** | **95% CI :LL to UL** |  |  | **Value** | **95% CI :LL to UL** | **Value** | | **95% CI :LL to UL** |
| 60 | L_Kynurenine | 0.461 | 0.335 to 0.587 | 0.535 | - |  |  |  | |  |
| 61 | L_Methionine | 0.187 | 0.092 to 0.283 | <0.001 | - |  |  |  | |  |
| 62 | L_Norleucine | 0.133 | 0.054 to 0.211 | <0.001 | - |  |  |  | |  |
| 63 | L_Phenylalanine | 0.403 | 0.277 to 0.530 | 0.126 | - |  |  |  | |  |
| 64 | L_Proline | 0.310 | 0.198 to 0.423 | 0.003 | - |  |  |  | |  |
| 65 | L_Tryptophan | 0.048 | 0.003 to 0.094 | <0.001 | - |  |  |  | |  |
| 66 | L_Valine | 0.269 | 0.163 to 0.375 | <0.001 | - |  |  |  | |  |
| 67 | m_Coumaric_acid | 0.178 | 0.088 to 0.267 | <0.001 | - |  |  |  | |  |
| 68 | N_Acetylputrescine | 0.578 | 0.455 to 0.701 | 0.220 | - |  |  |  | |  |
| 69 | N_Acetylserotonin | 0.517 | 0.390 to 0.643 | 0.795 | - |  |  |  | |  |
| 70 | N_Methylhydantoin | 0.049 | 0.007 to 0.090 | <0.001 | - |  |  |  | |  |
| 71 | Niacinamide | 0.299 | 0.179 to 0.419 | 0.001 | - |  |  |  | |  |
| 72 | Normetanephrine | 0.217 | 0.116 to 0.318 | <0.001 | - |  |  |  | |  |
| 73 | Nutriacholic_acid | 0.301 | 0.191 to 0.411 | 0.002 | - |  |  |  | |  |
| 74 | o_Tyrosine | 0.484 | 0.360 to 0.609 | 0.805 | - |  |  |  | |  |
| 75 | Oxalacetic_acid | 0.127 | 0.040 to 0.215 | <0.001 | - |  |  |  | |  |
| 76 | Oxypurinol | 0.176 | 0.084 to 0.268 | <0.001 | - |  |  |  | |  |
| 77 | Pantothenic_acid | 0.279 | 0.166 to 0.393 | <0.001 | - |  |  |  | |  |
| 78 | Paracetamol_sulfate | 0.836 | 0.660 to 1.000 | 0.002 | 652.5 | 81% | 65.4% to 90.9% | 88.9% | | 50.7% to 99.4% |
| 79 | Paraxanthine | 0.137 | 0.058 to 0.217 | <0.001 | - |  |  |  | |  |
| 80 | PC_16_0_16_0 | 0.523 | 0.397 to 0.649 | 0.722 | - |  |  |  | |  |
| 81 | PC_18_1_9Z__18_1_9Z | 0.338 | 0.220 to 0.455 | 0.010 | - |  |  |  | |  |
| 82 | Phenylpropiolic_acid | 0.153 | 0.069 to 0.238 | <0.001 | - |  |  |  | |  |
| 83 | Phosphoric_acid | 0.209 | 0.115 to 0.304 | <0.001 | - |  |  |  | |  |
| 84 | Pipecolic_acid | 0.328 | 0.212 to 0.444 | 0.006 | - |  |  |  | |  |
| 85 | Propanal | 0.459 | 0.334 to 0.584 | 0.515 | - |  |  |  | |  |
| 86 | Pyridoxal_5__phosphate | 0.443 | 0.320 to 0.566 | 0.369 | - |  |  |  | |  |
| 87 | Pyroglutamic_acid | 0.514 | 0.389 to 0.638 | 0.826 | - |  |  |  | |  |
| 88 | Quinaldic_acid *(cannot be computed)* |  |  |  |  |  |  |  | |  |
| 89 | Sepiapterin | 0.323 | 0.206 to 0.439 | 0.005 | - |  |  |  | |  |
| 90 | Serotonin | 0.035 | 0.000 to 0.091 | <0.001 | - |  |  |  | |  |
| **No** | **Clinical /Metabolite test** | **AUC** | | **P-value** | **Cut-off*** | **Sensitivity** | | | **Specificity** | |
|  |  | **Value** | **95% CI :LL to UL** |  |  | **Value** | **95% CI :LL to UL** | **Value** | | **95% CI :LL to UL** |
| 91 | Sphingosine | 0.493 | 0.366 to 0.621 | 0.916 | - |  |  |  | |  |
| 92 | Succinic_acid | 0.177 | 0.082 to 0.272 | <0.001 | - |  |  |  | |  |
| 93 | Succinylacetone | 0.671 | 0.556 to 0.786 | 0.007 | - |  |  |  | |  |
| 94 | Thyroxine | 0.314 | 0.190 to 0.437 | 0.004 | - |  |  |  | |  |
| 95 | Trimethylamine | 0.353 | 0.236 to 0.470 | 0.020 | - |  |  |  | |  |
| 96 | Urea | 0.371 | 0.250 to 0.492 | 0.041 | - |  |  |  | |  |
| 97 | Ureidosuccinic_acid | 0.370 | 0.251 to 0.489 | 0.039 | - |  |  |  | |  |
| 98 | Uric_acid | 0.230 | 0.127 to 0.333 | <0.001 | - |  |  |  | |  |
| 99 | Uridine | 0.379 | 0.259 to 0.500 | 0.055 | - |  |  |  | |  |

**Optimal cut-off values were calculated only for tests showing high/moderate accuracy levels in predicting severity of COVID-19 (AUC > 0.70)*
