# Supplementary material for: Plasma metabolomics profiling identifies new predictive biomarkers for disease severity in COVID-19 patients
Source: PLoS One. 2023 Aug 10;18(8):e0289738. doi: 10.1371/journal.pone.0289738 (PMC10414581; doi:10.1371/journal.pone.0289738)
Supplement: S2 Table — (DOCX) [file pone.0289738.s002.docx]

Supplementary Table 2. Correlations between Ferritin and the different metabolites

| **No** | **Metabolite test** | **n** | **Spearman Correlation coefficient ( ρ )** | **P-value** | **95% Confidence Interval**  **LL to UL** |
| --- | --- | --- | --- | --- | --- |
| 1 | K_1_3_Dimethyluric_acid | 71 | -0.570 | <0.001 | -0.712 to -0.382 |
| 2 | K_1_Methyladenosine | 82 | -0.223 | 0.044 | -0.425 to 0.000 |
| 3 | K_2_5_Furandicarboxylic_acid | 85 | -0.500 | <0.001 | -0.649 to -0.316 |
| 4 | K_2_Pyrrolidinone | 85 | -0.020 | 0.857 | -0.238 to 0.200 |
| 5 | K_3_4_5_Trimethoxycinnamic_acid | 85 | -0.325 | 0.002 | -0.508 to -0.114 |
| 6 | K_3_5_Dimethoxyphenol | 85 | 0.034 | 0.757 | -0.187 to 0.251 |
| 7 | K_3_Indolepropionic_acid | 75 | -0.404 | <0.001 | -0.582 to -0.188 |
| 8 | K_3_Methylindole | 85 | -0.654 | <0.001 | -0.764 to -0.508 |
| 9 | K_3_Methylxanthine | 85 | -0.624 | <0.001 | -0.742 to -0.469 |
| 10 | K_4_Aminophenol | 85 | 0.573 | <0.001 | 0.405 to 0.704 |
| 11 | K_5_Hydroxy_L_tryptophan | 85 | -0.681 | <0.001 | -0.783 to -0.542 |
| 12 | K_5_Hydroxyindoleacetic_acid | 85 | -0.164 | 0.134 | -0.370 to 0.058 |
| 13 | K_9_Methyluric_acid | 80 | -0.528 | <0.001 | -0.673 to -0.343 |
| 14 | Acetaminophen | 78 | 0.670 | <0.001 | 0.521 to 0.780 |
| 15 | Acetaminophen_glucuronide | 48 | 0.159 | 0.281 | 0.140 to 0.431 |
| 16 | Acetic_acid | 85 | 0.170 | 0.119 | -0.051 to 0.375 |
| 17 | Acetone | 85 | -0.131 | 0.231 | -0.341 to 0.091 |
| 18 | Adenosine_monophosphate | 85 | -0.428 | <0.001 | -0.592 to -0.231 |
| 19 | Allantoin | 85 | -0.096 | 0.381 | -0.309 to 0.126 |
| 20 | Alpha_ketoisovaleric_acid | 85 | -0.666 | <0.001 | -0.772 to -0.523 |
| 21 | Alpha_N_phenylacetul_L_glutamine | 85 | 0.035 | 0.749 | -0.185 to 0.252 |
| 22 | Aniline | 85 | -0.122 | 0.265 | -0.333 to 0.100 |
| 23 | Aspartame | 85 | 0.044 | 0.692 | -0.177 to 0.260 |
| 24 | Azelaic_acid | 84 | -0.333 | 0.002 | -0.516 to -0.122 |
| 25 | Benzaldehyde | 85 | -0.147 | 0.178 | -0.355 to 0.074 |
| 26 | Benzocaine | 85 | 0.018 | 0.870 | -0.202 to 0.236 |
| 27 | Benzoic_acid | 85 | -0.418 | <0.001 | -0.584 to -0.219 |
| 28 | Cadaverine | 85 | -0.251 | 0.020 | -0.446 to -0.034 |
| **No** | **Clinical /Metabolite test** | **n** | **Spearman Correlation coefficient ( ρ )** | **P-value** | **95% Confidence Interval**  **LL to UL** |
| 29 | Caffeine | 85 | -0.442 | <0.001 | -0.603 to -0.247 |
| 30 | Chlorpheniramine | 84 | -0.129 | 0.243 | -0.340 to 0.094 |
| 31 | Cinnamic_acid | 85 | -0.155 | 0.157 | -0.362 to 0.067 |
| 32 | Cis_Aconitic_acid | 85 | -0.397 | <0.001 | -0.567 to -0.194 |
| 33 | Cortisol | 85 | -0.103 | 0.349 | -0.315 to 0.119 |
| 34 | Creatine | 85 | -0.501 | <0.001 | -0.649 to -0.316 |
| 35 | Cytosine | 84 | 0.416 | <0.001 | 0.215 to 0.583 |
| 36 | Deoxycholic_acid_glycine_conjugate | 80 | -0.330 | 0.003 | -0.517 to -0.112 |
| 37 | DL_2_aminooctanoic_acid | 85 | -0.534 | <0.001 | -0.674 to -0.357 |
| 38 | Elaidic_acid | 85 | 0.249 | 0.022 | 0.031 to 0.444 |
| 39 | Ethanolamine | 85 | -0.319 | 0.003 | -0.503 to -0.107 |
| 40 | Glucosamine | 84 | -0.188 | 0.086 | -0.393 to 0.033 |
| 41 | Glycerophosphocholine | 85 | -0.611 | <0.001 | -0.732 to -0.452 |
| 42 | Glycine | 85 | 0.222 | 0.041 | 0.003 to 0.421 |
| 43 | Glycocholic_acid | 83 | -0.192 | 0.081 | -0.397 to 0.031 |
| 44 | Guanidine | 85 | -0.119 | 0.280 | -0.329 to 0.103 |
| 45 | Hippuric_acid | 77 | -0.400 | <0.001 | -0.577 to -0.187 |
| 46 | Homoveratric_acid | 85 | -0.038 | 0.729 | -0.255 to 0.183 |
| 47 | Hypoxanthine | 84 | -0.435 | <0.001 | -0.598 to -0.237 |
| 48 | Indole | 84 | -0.670 | <0.001 | -0.776 to -0.527 |
| 49 | Indoleacetic_acid | 85 | -0.477 | <0.001 | -0.630 to -0.288 |
| 50 | Indolelactic_acid | 83 | -0.563 | <0.001 | -0.698 to -0.390 |
| 51 | Inosinic_acid | 84 | -0.574 | <0.001 | -0.705 to -0.405 |
| 52 | Isobutyric_acid | 85 | 0.276 | 0.010 | 0.061 to 0.467 |
| 53 | Isovalerylcarnitine | 85 | -0.111 | 0.311 | -0.323 to 0.111 |
| 54 | Kynurenic_acid | 85 | -0.238 | 0.028 | -0.434 to -0.020 |
| 55 | L_Acetylcarnitine | 85 | -0.135 | 0.216 | -0.344 to 0.086 |
| 56 | L_Arginine | 85 | -0.599 | <0.001 | -0.723 to -0.437 |
| 57 | L_Carnitine | 85 | -0.334 | 0.002 | -0.516 to -0.124 |
| 58 | L_Glutamine | 84 | 0.053 | 0.633 | -0.170 to 0.270 |
| 59 | L_Histidine | 85 | -0.537 | <0.001 | -0.676 to -0.360 |
| **No** | **Clinical /Metabolite test** | **n** | **Spearman Correlation coefficient ( ρ )** | **P-value** | **95% Confidence Interval**  **LL to UL** |
| 60 | L_Kynurenine | 85 | -0.071 | 0.518 | -0.286 to 0.150 |
| 61 | L_Methionine | 85 | -0.360 | <0.001 | -0.537 to -0.153 |
| 62 | L_Norleucine | 85 | -0.517 | <0.001 | -0.661 to -0.335 |
| 63 | L_Phenylalanine | 85 | -0.047 | 0.672 | -0.263 to 0.174 |
| 64 | L_Proline | 85 | -0.249 | 0.022 | -0.444 to -0.032 |
| 65 | L_Tryptophan | 85 | -0.642 | <0.001 | -0.755 to -0.492 |
| 66 | L_Valine | 85 | -0.354 | <0.001 | -0.532 to -0.146 |
| 67 | m_Coumaric_acid | 85 | -0.472 | <0.001 | -0.626 to -0.281 |
| 68 | N_Acetylputrescine | 84 | 0.130 | 0.240 | -0.094 to 0.340 |
| 69 | N_Acetylserotonin | 82 | 0.035 | 0.758 | -0.190 to 0.256 |
| 70 | N_Methylhydantoin | 85 | -0.597 | <0.001 | -0.722 to -0.435 |
| 71 | Niacinamide | 85 | -0.306 | 0.004 | -0.492 to -0.093 |
| 72 | Normetanephrine | 84 | -0.438 | <0.001 | -0.601 to -0.241 |
| 73 | Nutriacholic_acid | 85 | -0.297 | 0.006 | -0.485 to -0.083 |
| 74 | o_Tyrosine | 85 | -0.106 | 0.333 | -0.318 to 0.116 |
| 75 | Oxalacetic_acid | 70 | -0.553 | <0.001 | -0.701 to -0.360 |
| 76 | Oxypurinol | 82 | -0.479 | <0.001 | -0.634 to -0.286 |
| 77 | Pantothenic_acid | 85 | -0.353 | <0.001 | -0.531 to -0.145 |
| 78 | Paracetamol_sulfate | 51 | 0.237 | 0.095 | -0.050 to 0.487 |
| 79 | Paraxanthine | 85 | -0.503 | <0.001 | -0.651 to -0.3119 |
| 80 | PC_16_0_16_0 | 83 | 0.111 | 0.316 | -0.113 to 0.325 |
| 81 | PC_18_1_9Z__18_1_9Z | 85 | -0.215 | 0.048 | -0.414 to 0.005 |
| 82 | Phenylpropiolic_acid | 85 | -0.501 | <0.001 | -0.649 to -0.316 |
| 83 | Phosphoric_acid | 85 | -0.414 | <0.001 | -0.580 to -0.214 |
| 84 | Pipecolic_acid | 85 | -0.281 | 0.009 | -0.471 to -0.066 |
| 85 | Propanal | 85 | -0.080 | 0.469 | -0.294 to 0.142 |
| 86 | Pyridoxal_5__phosphate | 85 | -0.087 | 0.430 | -0.300 to 0.135 |
| 87 | Pyroglutamic_acid | 85 | 0.019 | 0.861 | -0.201 to 0.237 |
| 88 | Quinaldic_acid *(cannot be computed)* | 14 | 0.086 | 0.771 | -0.480 to 0.601 |
| 89 | Sepiapterin | 83 | -0.230 | 0.037 | -0.430 to -0.008 |
| 90 | Serotonin | 37 | -0.697 | <0.001 | -0.836 to -0.474 |
| **No** | **Clinical /Metabolite test** | **n** | **Spearman Correlation coefficient ( ρ )** | **P-value** | **95% Confidence Interval**  **LL to UL** |
| 91 | Sphingosine | 85 | 0.053 | 0.627 | -0.168 to 0.270 |
| 92 | Succinic_acid | 79 | -0.465 | <0.001 | -0.626 to -0.265 |
| 93 | Succinylacetone | 85 | 0.257 | 0.018 | 0.040 to 0.451 |
| 94 | Thyroxine | 82 | -0.270 | 0.014 | -0.465 to -0.050 |
| 95 | Trimethylamine | 85 | -0.118 | 0.282 | -0.329 to 0.104 |
| 96 | Urea | 85 | -0.273 | 0.012 | -0.464 to -0.057 |
| 97 | Ureidosuccinic_acid | 85 | -0.132 | 0.229 | -0.341 to 0.090 |
| 98 | Uric_acid | 85 | -0.506 | <0.001 | -0.653 to -0.323 |
| 99 | Uridine | 85 | -0.096 | 0.384 | -0.308 to 0.126 |
