# Supplementary material for: Plasma metabolomics profiling identifies new predictive biomarkers for disease severity in COVID-19 patients
Source: PLoS One. 2023 Aug 10;18(8):e0289738. doi: 10.1371/journal.pone.0289738 (PMC10414581; doi:10.1371/journal.pone.0289738)
Supplement: Attachment — Submitted filename: Review comments.docx [file pone.0289738.s003.docx]

High importance overall comments

Required

1. As briefly mentioned at the end of the discussion session this study requires a validation. No test / validation population was curved out from the initial cohort and all reported results calculated on the training data. As such they are most likely unreliably optimistic. This is hinted by the unusually high AUC values. I would strongly recommend that the proposed model(s) performance is validated in a new cohort prior to publication.
2. As only few metabolites resulting from LC-MS were retained for the models I recommend that prior to publication the identification of those is validated against a standard. This will give the high confidence level (MSI 1) required to be of use to the scientific community. Do not rely solely on match to public libraries. More specifically in the case of COVID-19 patients a lot of cytosine containing compounds are elevated. For many of those compounds the cytosine sub-structure tends to break easily at the MS source resulting in multitude of cytosine events in an acquired TIC. Each of those events will have a good match to cytosine based on MS/MS, but not on RT. Please make sure that the metabolic feature you selected for you model overlap in RT with a cytosine standard for your method i.e., column and LC-MS conditions. I have a high confidence that it is not cytosine, but a source fragmentation artifact of more complex molecule that contains a cytosine substructure.
3. I strongly believe that predictive models should not rely on detecting medication (K_4_aminophenol, acetaminophen) in patient’s blood. This is highly subjective to patient’s lifestyle choices and hospital practices.
4. The study states in the abstract that “almost all such models, which relied on serum/plasma biomarkers, clinical data or a combination of both, were subsequently deemed as cumbersome, inadequate and/or subject to bias”. However, very few predictive models of COVID severity were discussed in this manuscript. I don’t believe that this statement was well defended. A more thorough discussion on the alignment / misalignment of this study finding with previous models will be beneficial to the community.

In addition of this I have made non-exhaustive inline comments with importance noted as high or low.

Data availability. (high) I could not find the study ID 3469 on <https://www.metabolomicsworkbench.org/>. The highest ID number available there is ST002303.

Page 4, line 67-70. (high) The aggressiveness in this sentence is going too far, specifically the usage of ‘utter incompetence’.

Page 4, line 70-72. (low) I would argue that the scientific community response was quite good, especially when it comes to speed of developing testing abilities and vaccines.

Page 5, line 100-103. (low) It seems here that you contradict yourselves between the 2 sentences. First you state that it’s predictable that metabolic response varies widely because of lifestyle (which usually includes diet), comorbidities, medication etc. If true, this will make metabolic profiles a poor base of prediction / diagnosis, as all the combinations of those factors are way too large to be accounted for as separate groups. Then in the next sentence you defend the seemingly similar response of a host to infection that justifies the use for prognosis prediction.

In my opinion biological response to stressors can often be surprisingly predictable, despite lifestyle, comorbidities and other differences and therefore metabolic profiles are a good foundation for diagnosis / predictions. You may want to revisit the sentence on line 100-102 to reconcile the contradiction.

Page 7, line 148. (low) Tiny change to facilitate the reading, it’s best to respect the order of the volumes when giving the description. 3:1 (90 uL methanol: 30 uL sample) rather than the current 3:1 (30 uL sample: 90 uL methanol)

Page 8, line 150- 152. (low) One would expect something solid and reproducible here and the use of the word ‘usually’ gives the wrong impression. It’s not clear what exactly you mean by the volume is transferred three times? If you mean that you get 90 uL of supernatant for 30 uL of sample (i.e., 3 times the initial volume) – than just say that: 90 uL of supernatant was transferred to a new microcentrifuge tube.

Page 8, line 152-153. (high) Please provide what solvent was used to rehydrate the samples.

Page 8, line 154. (low) Please provide the LC and MS brands.

Page 8, line 154. (low) Two publications have been cited here as reference to more detailed description of the LC-MS method employed. However, neither of the cited publications contain more details, they have actually less details than further provided in this manuscript. If you insist to cite your group publications, please rephrase this to ‘as similar to’ rather than ‘as described’. Otherwise, you are wasting readers time to go look for details.

Page 8, line 160. (low) Interesting choice of 8C for the autosampler temperature … why not the most common 4C? Have you compared the sample stability throughout the run when held at 8C vs 4C?

Page 8, line 167. (low) ‘software provided available’ this is confusing.

Page 8, line 170. (high) “3 (of 12) samples (per cell type)” This is confusing. Of course, you want to retain only metabolic features that are consistent in certain number of samples – usually all QC samples, but what are cell types? Why 3 out of 12? Where there QCs? I didn’t see a mention to pooled QC anywhere.

Methods section: (high) There is no mention of use of QC pool samples, neither randomization. Is this missing in the text only or was not performed in the study?

Page 8, line 166 – Page 9 line 181.

(low) It seems that you have performed untargeted LC-MS even though the word untargeted is not mentioned anywhere else than in the keywords. However, only 99 metabolites were measured (results section). This is an unusually low number for untargeted LC-MS. Is seems that you discarded all features that were not confidently identified by the software. It would be better to state this more clearly in the methods. Also, it would be best to provide the score cutoffs that you have used on IDs.

(high) Finally, as not all metabolites have been matched against MS/MS and RT please provide level of identification confidence in results section for each metabolite (MS/MS alone, MS/MS & RT). Please follow the standards described in Sumner, Lloyd W., et al. "Proposed minimum reporting standards for chemical analysis." *Metabolomics* 3.3 (2007): 211-221.

Page 8, line 169: (high) “Feature quantitation”: the use of quantitation here is misleading as this term is very specific to quantitative LC-MS and it does not seem that you have performed quantitative study.

Page 9, line 182-184. (low) Please add citation to MetaboAnalyst missing and to pathway enrichment analysis.

Page 10, line 216- Page 11, line 221. (high) Why using a cut-off / scoring system instead of multiple predictor models? This makes you lose the effect strength of those predictors. Please justify your choice, why is this better?

Page 11, line 237. (high) Please provide all units in table 1. Example CRP should have a unit.

Page 14, line 301 – 305. (high) What are the units of this cut-off values for the metabolites? How this, if even possible at all, could be used by another lab? Are those standardized ion counts values?

Page 15, line 322. (high) The use of medication such as Acetaminophen and K_4_Aminophenol (degradation product of paracetamol) in a predictive model is questionable. Probably more easily reported by the patient and medical staff than measured in blood. Why not submitting only one model? The two models make the results harder to follow. Consider reporting only the model that do not contain medications.

Page 18, line 385. (low) “…other reports is the verified the lower level of” review sentence grammar.

Page 18, line 387-396. (high) The review manuscript cited here concludes the change in KYN/TRP ratio is resulting from an increase in kynurenine and decrease in tryptophan which only partially agrees with your results. Your data shown no change in kynurenine in severe cases. Please add KYN/TRP ratio analysis. Misalignment in results is also valuable.

**Tables**

Overall. In general table notes lack of details. There is very little description and tables should be more self-explanatory, i.e., one should be able to understand even if this is the only thing that they read in the manuscript.

Page 2, Table 1- (high) The column Severity of Covid-19 separated in 2 groups Asymptomatic/Mild n(%) and Severe n (%) actually refers to the mean not the count (n) in some clinical lab tests. This need to be made clearer. Please add all units too, when relevant.

Page 4 Table 2: (high) Please consider using the same test for all features. Mann Whitney U test could be used in all cases. Also consider standardizing the data prior to the test.

Page 8, Table 3: (high) As this is an important table for the results please add a more stand-alone description, covering again how AUC and cut-offs were calculated.

In general, it’s kind of unclear what method was used to calculate a particular AUC. Some one-predictor AUCs are so high that they seem unrealistic.
