# Supplementary material for: Plasma metabolomics profiling identifies new predictive biomarkers for disease severity in COVID-19 patients
Source: PLoS One. 2023 Aug 10;18(8):e0289738. doi: 10.1371/journal.pone.0289738 (PMC10414581; doi:10.1371/journal.pone.0289738)
Supplement: Attachment — Submitted filename: Request_revision_May_2023_MH.docx [file pone.0289738.s004.docx]

**Plasma metabolomics profiling identifies new predictive biomarkers for disease severity in COVID-19 patients**

Nelson C. Soares^1,2‡^, Amal Hussein^3‡^, Jibran Sualeh Muhammad^1,4^, Mohammad H. Semreen^1,2^, Gehad ElGhazali^6^, Mawieh Hamad^1,5^*

Dear Dr. Hamad,

We've checked your submission and before we can proceed, we need you to address the following issues:

**1. Please note that funding information should not appear in the Acknowledgments section/Funding section or Any other areas of your Manuscript**. We will only publish funding information present in the Funding Statement section of the online submission form. Please remove any funding-related text from the manuscript.

**R.1 Acknowledgments section -** *This work was supported by research grant CoV19-0305 (MH), seed grant 2001110138, University of Sharjah, UAE. This research is part of the -Human Disease Biomarkers Discovery Research Group-study. The authors wish to acknowledge the generous support of the Research Institute for Medical and Health Sciences, University of Sharjah UAE*. *The funders had no role in study design, data collection and analysis, decision to publish, or preparation of the manuscript.*

**Has been removed as suggested.**

**2. If you are reporting a retrospective study of medical records or archived samples, please ensure that you have discussed whether all data were fully anonymized before you accessed them and/or whether the IRB or ethics committee waived the requirement for informed consen**t. If patients provided informed written consent to have data from their medical records used in research, please include this information.

**R.2** This retrospective study used archived samples collected from patients in Sheikh Khalifa Medical City (SKMC), Abu Dhabi, UAE between March 20 until July 17, 2020. In the materials and methods section, sample collection subheading, we stated “*The study was jointly approved by the Ministry of Health, Abu Dhabi and Dubai Health Authority (DOH/CVDC/2020/1949) on the understanding that samples will be number-coded to hide patient’s identity, that no personal information will be shared with a third party and that no sample analysis can be performed by entities other than the Research Institute of Medical and Health Sciences (RIMHS), the University of Sharjah (UOS) without prior written approval*”. To further clarify this issue, the following statement was added towards the end of the sample collection subheading: No informed consent was required as per the ethical approval decision (*DOH/CVDC/2020/1949*); in compliance with the said decision, all samples were fully anonymized before accessing or receiving them.

**3. Thank you for stating the following financial disclosure:**
 
"This work was supported by research grant CoV19-0305 (MH), seed grant 2001110138 (NCS), University of Sharjah, UAE. This research is part of the -Human Disease Biomarkers Discovery Research Group-study. The authors wish to acknowledge the generous support of the Research Institute for Medical and Health Sciences, University of Sharjah UAE"       
 
Please state what role the funders took in the study.  If the funders had no role, please state: "The funders had no role in study design, data collection and analysis, decision to publish, or preparation of the manuscript."

If this statement is not correct you must amend it as needed.

Please include this amended Role of Funder statement in your cover letter; we will change the online submission form on your behalf

**R.3** **The statement:** "*The funders had no role in study design, data collection and analysis, decision to publish, or preparation of the manuscript*."


4. **Please amend your list of authors on the manuscript to ensure that each author is linked to an affiliation**.
We note that you have included affiliation numbers 1,2,3, 4, 5 and 6 however only affiliations 1,2, 3, 5 and 6 have authors linked to them.  Please amend affiliation 4 to link an author to it or remove if added in error.

**R4** This has been changed accordingly

5. **Please include your tables as part of your main manuscript and remove the individual files**. Please note that supplementary tables (should remain/ be uploaded) as separate "Supporting Information" files

**R.5** is has been changed accordingly- note check supplementary tables at the time of resubmission
